# Supplementary material for: Assessing the association between air pollution and child development in São Paulo, Brazil
Source: PLoS One. 2022 May 13;17(5):e0268192. doi: 10.1371/journal.pone.0268192 (PMC9106172; doi:10.1371/journal.pone.0268192)
Supplement: S1 Table — (DOCX) [file pone.0268192.s001.docx]

Supporting information

S1 Table Participants’ characteristics at 72-month follow-up

| Participants’ characteristics given as N* (Percentage) or Mean ± SD** | | | | |
| --- | --- | --- | --- | --- |
| Female gender | | | 558 | (48.82) |
| Age in months | | | 76 | ± 5.2 |
| Child’s skin-color | | White | 517 | (45.23) |
|  |  | Mixed | 581 | (50.83) |
|  |  | Black | 42 | (3.67) |
|  |  | Others | 3 | (0.26) |
| Low weight at birth (<2500g) | | | 74 | (6.47) |
| Pre-term gestational length | | | 81 | (7.1) |
| Delivery type | | Regular | 545 | (47.68) |
|  |  | Caesarean | 423 | (37.01) |
|  |  | Forceps | 175 | (15.31) |
| Mother’s age at delivery | | ≤19 | 172 | (15.05) |
|  |  | 20-29 | 609 | (53.28) |
|  |  | ≥30 | 362 | (31.67) |
| Mother’s skin-color | | White | 667 | (58.36) |
|  |  | Mixed | 428 | (37.45) |
|  |  | Black | 47 | (4.11) |
|  |  | Others | 1 | (0.09) |
| Mother having depression | | | 500 | (43.74) |
| Caregiver’s age | | | 34.97 | ±9.8 |
| Caregiver is married or live with a partner | | | 729 | (64.63) |
| Caregiver relation to the child | Mother | | 901 | (79.10) |
|  | Grandmother | | 116 | (10.18) |
|  | Other family member | | 101 | (8.87) |
|  | No relation | | 21 | (1.84) |
| Caregiver’s highest grade completed | | None | 23 | (2.02) |
|  |  | Elementary | 342 | (30.08) |
|  |  | Middle | 656 | (57.7) |
|  |  | Upper | 116 | (10.2) |
| Educational level of the head of household | | Illiterate | 30 | (2.65) |
|  |  | Elementary incomplete | 280 | (24.69) |
|  |  | Elementary | 311 | (27.43) |
|  |  | Middle | 465 | (41.01) |
|  |  | Upper | 48 | (4.23) |
| Household size | | | 4.5 | ±1.5 |
| Households getting financial support | | | 272 | (23.8) |
| Socio economic status in Brazilian classification | | A | 4 | (0.35) |
|  |  | B1 | 18 | (1.58) |
|  |  | B2 | 99 | (8.68) |
|  |  | C1 | 367 | (32.19) |
|  |  | C2 | 492 | (43.16) |
|  |  | D-E | 160 | (14.04) |
| Number of stimulating activities done with the child in the three days before assessment | | 0 | 23 | (2.02) |
|  |  | 1 | 54 | (4.73) |
|  |  | 2 | 138 | (12.09) |
|  |  | 3 | 161 | (14.11) |
|  |  | 4 | 180 | (15.78) |
|  |  | 5 | 198 | (17.35) |
|  |  | 6 | 387 | (33.92) |
