# Supplementary material for: Assessing the association between air pollution and child development in São Paulo, Brazil
Source: PLoS One. 2022 May 13;17(5):e0268192. doi: 10.1371/journal.pone.0268192 (PMC9106172; doi:10.1371/journal.pone.0268192)
Supplement: S2 Table — (DOCX) [file pone.0268192.s002.docx]

S2 Table Participants’ characteristics at birth vs. at 72-month follow-up

| Sample characteristics at birth vs. sample at 72-month follow-up | | | | | |
| --- | --- | --- | --- | --- | --- |
| Variable | | **At birth** | | **At 6y follow-up** | |
|  |  | Absolute numbers | Percentage (%) | Absolute numbers | Percentage (%) |
| Child’s gender | **Male** | 3092 | 49.83 | 800 | 51.35 |
|  | **Female** | 3113 | 50.17 | 758 | 48.65 |
|  | **N** | 6205 |  | 1558 |  |
| Child’s  skin-color | **White** | 2878 | 46.37 | 699 | 44.87 |
|  | **Mixed** | 3101 | 49.97 | 798 | 51.22 |
|  | **Black** | 213 | 3.43 | 57 | 3.66 |
|  | **Indigenous** | 9 | 0.15 | 3 | 0.19 |
|  | **Yellow** | 5 | 0.08 | 1 | 0.06 |
|  | **N** | 6206 |  | 1558 |  |
| Type of delivery | **Regular** | 2974 | 47.91 | 728 | 46.73 |
|  | **Caesarean** | 2316 | 37.31 | 584 | 37.48 |
|  | **Forceps** | 917 | 14.77 | 246 | 15.79 |
|  | **N** | 6207 |  | 1558 |  |
| Gestational length | **pre-term** | 483 | 7.78 | 109 | 7.00 |
|  | **post-term** | 16 | 0.26 | 6 | 0.39 |
|  | **full-term** | 5708 | 91.96 | 1443 | 92.62 |
|  | **N** | 6207 |  | 1558 |  |
| Weight at birth | **normal (≥2500g)** | 465 | 7.49 | 103 | 6.61 |
|  | **low <2500 g** | 5744 | 92.51 | 1455 | 93.39 |
|  | **N** | 6209 |  | 1558 |  |
| Mother’s age | **≤19** | 1057 | 17.02 | 246 | 15.79 |
|  | **20-29** | 3349 | 53.94 | 825 | 52.95 |
|  | **≥30** | 1803 | 29.04 | 487 | 31.26 |
|  | **N** | 6209 |  | 1558 |  |
| Mother’s skin-color | **white** | 3784 | 60.97 | 910 | 58.41 |
|  | **mixed** | 2159 | 34.79 | 581 | 37.29 |
|  | **black** | 257 | 4.14 | 66 | 4.24 |
|  | **indigenous** | 1 | 0.02 | 0 | 0 |
|  | **yellow** | 5 | 0.08 | 1 | 0.06 |
|  | **N** | 6206 |  | 1558 |  |
