# Supplementary material for: Assessing the association between air pollution and child development in São Paulo, Brazil
Source: PLoS One. 2022 May 13;17(5):e0268192. doi: 10.1371/journal.pone.0268192 (PMC9106172; doi:10.1371/journal.pone.0268192)
Supplement: S3 Table — (DOCX) [file pone.0268192.s003.docx]

S3 Table Sensitivity analysis without outliers

| Association of NO_2_ exposure as continuous [μg/m^3^] with IDELA as z-score without values lower than -3.5 and with CBCL as z-score without values higher than 3.75, unadjusted and adjusted* models. Results are expressed per 10 μg/m^3^. | | | | | | |
| --- | --- | --- | --- | --- | --- | --- |
|  | **Unadjusted** |  | **N** | **Adjusted** |  | **N** |
| IDELA z-score | β (95% CI) | p-value |  | β (95% CI) | p-value |  |
|  | -0.03 (-0.17;0.11) | 0.65 | 1132 | -0.04 (-0.18;0.11) | 0.63 | 1087 |
| CBCL z-score |  |  |  |  |  |  |
|  | 0.01 (-0.08;0.11) | 0.77 | 1137 | 0.03 (-0.07;0.12) | 0.58 | 1093 |
| * IDELA and CBCL models adjusted for: child gender, child age in months, child skin-color, birthweight, gestational length, delivery type, mother’s age at delivery, mother’s skin-color, maternal depression, caregiver’s marital status, caregiver’s relation to the child, caregiver’s age, highest school grades of caregiver, highest school grade of household head, household size, financial support, socio-economic status, and home stimulation score. | | | | | | |
| IDELA = International Development and Early Learning Assessment; CBCL = Child Behavior Checklist; CI = Confidence interval | | | | | | |
