# Supplementary material for: Assessing the association between air pollution and child development in São Paulo, Brazil
Source: PLoS One. 2022 May 13;17(5):e0268192. doi: 10.1371/journal.pone.0268192 (PMC9106172; doi:10.1371/journal.pone.0268192)
Supplement: S1 File — (DOCX) [file pone.0268192.s005.docx]

# Supporting Information

A Participants’ characteristics

A.1 Participants’ characteristics at 72-month follow-up

| Participants’ characteristics given as N* (Percentage) or Mean ± SD** | | | | |
| --- | --- | --- | --- | --- |
| Female gender | | | 558 | (48.82) |
| Age in months | | | 76 | ± 5.2 |
| Child’s skin-color | | White | 517 | (45.23) |
|  |  | Mixed | 581 | (50.83) |
|  |  | Black | 42 | (3.67) |
|  |  | Others | 3 | (0.26) |
| Low weight at birth (<2500g) | | | 74 | (6.47) |
| Pre-term gestational length | | | 81 | (7.1) |
| Delivery type | | Regular | 545 | (47.68) |
|  |  | Caesarean | 423 | (37.01) |
|  |  | Forceps | 175 | (15.31) |
| Mother’s age at delivery | | ≤19 | 172 | (15.05) |
|  |  | 20-29 | 609 | (53.28) |
|  |  | ≥30 | 362 | (31.67) |
| Mother’s skin-color | | White | 667 | (58.36) |
|  |  | Mixed | 428 | (37.45) |
|  |  | Black | 47 | (4.11) |
|  |  | Others | 1 | (0.09) |
| Mother having depression | | | 500 | (43.74) |
| Caregiver’s age | | | 34.97 | ±9.8 |
| Caregiver is married or live with a partner | | | 729 | (64.63) |
| Caregiver relation to the child | Mother | | 901 | (79.10) |
|  | Grandmother | | 116 | (10.18) |
|  | Other family member | | 101 | (8.87) |
|  | No relation | | 21 | (1.84) |
| Caregiver’s highest grade completed | | None | 23 | (2.02) |
|  |  | Elementary | 342 | (30.08) |
|  |  | Middle | 656 | (57.7) |
|  |  | Upper | 116 | (10.2) |
| Educational level of the head of household | | Illiterate | 30 | (2.65) |
|  |  | Elementary incomplete | 280 | (24.69) |
|  |  | Elementary | 311 | (27.43) |
|  |  | Middle | 465 | (41.01) |
|  |  | Upper | 48 | (4.23) |
| Household size | | | 4.5 | ±1.5 |
| Households getting financial support | | | 272 | (23.8) |
| Socio economic status in Brazilian classification | | A | 4 | (0.35) |
|  |  | B1 | 18 | (1.58) |
|  |  | B2 | 99 | (8.68) |
|  |  | C1 | 367 | (32.19) |
|  |  | C2 | 492 | (43.16) |
|  |  | D-E | 160 | (14.04) |
| Number of stimulating activities done with the child in the three days before assessment | | 0 | 23 | (2.02) |
|  |  | 1 | 54 | (4.73) |
|  |  | 2 | 138 | (12.09) |
|  |  | 3 | 161 | (14.11) |
|  |  | 4 | 180 | (15.78) |
|  |  | 5 | 198 | (17.35) |
|  |  | 6 | 387 | (33.92) |

A.2 Participants’ characteristics at birth vs. at 72-month follow-up

| Sample characteristics at birth vs. sample at 72-month follow-up | | | | | |
| --- | --- | --- | --- | --- | --- |
| Variable | | **At birth** | | **At 6y follow-up** | |
|  |  | Absolute numbers | Percentage (%) | Absolute numbers | Percentage (%) |
| Child’s gender | **Male** | 3092 | 49.83 | 800 | 51.35 |
|  | **Female** | 3113 | 50.17 | 758 | 48.65 |
|  | **N** | 6205 |  | 1558 |  |
| Child’s  skin-color | **White** | 2878 | 46.37 | 699 | 44.87 |
|  | **Mixed** | 3101 | 49.97 | 798 | 51.22 |
|  | **Black** | 213 | 3.43 | 57 | 3.66 |
|  | **Indigenous** | 9 | 0.15 | 3 | 0.19 |
|  | **Yellow** | 5 | 0.08 | 1 | 0.06 |
|  | **N** | 6206 |  | 1558 |  |
| Type of delivery | **Regular** | 2974 | 47.91 | 728 | 46.73 |
|  | **Caesarean** | 2316 | 37.31 | 584 | 37.48 |
|  | **Forceps** | 917 | 14.77 | 246 | 15.79 |
|  | **N** | 6207 |  | 1558 |  |
| Gestational length | **pre-term** | 483 | 7.78 | 109 | 7.00 |
|  | **post-term** | 16 | 0.26 | 6 | 0.39 |
|  | **full-term** | 5708 | 91.96 | 1443 | 92.62 |
|  | **N** | 6207 |  | 1558 |  |
| Weight at birth | **normal (≥2500g)** | 465 | 7.49 | 103 | 6.61 |
|  | **low <2500 g** | 5744 | 92.51 | 1455 | 93.39 |
|  | **N** | 6209 |  | 1558 |  |
| Mother’s age | **≤19** | 1057 | 17.02 | 246 | 15.79 |
|  | **20-29** | 3349 | 53.94 | 825 | 52.95 |
|  | **≥30** | 1803 | 29.04 | 487 | 31.26 |
|  | **N** | 6209 |  | 1558 |  |
| Mother’s skin-color | **white** | 3784 | 60.97 | 910 | 58.41 |
|  | **mixed** | 2159 | 34.79 | 581 | 37.29 |
|  | **black** | 257 | 4.14 | 66 | 4.24 |
|  | **indigenous** | 1 | 0.02 | 0 | 0 |
|  | **yellow** | 5 | 0.08 | 1 | 0.06 |
|  | **N** | 6206 |  | 1558 |  |

B Sensitivity analysis

B.1 Sensitivity analysis without outliers

| Association of NO_2_ exposure as continuous [μg/m^3^] with IDELA as z-score without values lower than -3.5 and with CBCL as z-score without values higher than 3.75, unadjusted and adjusted* models. Results are expressed per 10 μg/m^3^. | | | | | | |
| --- | --- | --- | --- | --- | --- | --- |
|  | **Unadjusted** |  | **N** | **Adjusted** |  | **N** |
| IDELA z-score | β (95% CI) | p-value |  | β (95% CI) | p-value |  |
|  | -0.03 (-0.17;0.11) | 0.65 | 1132 | -0.04 (-0.18;0.11) | 0.63 | 1087 |
| CBCL z-score |  |  |  |  |  |  |
|  | 0.01 (-0.08;0.11) | 0.77 | 1137 | 0.03 (-0.07;0.12) | 0.58 | 1093 |
| * IDELA and CBCL models adjusted for: child gender, child age in months, child skin-color, birthweight, gestational length, delivery type, mother’s age at delivery, mother’s skin-color, maternal depression, caregiver’s marital status, caregiver’s relation to the child, caregiver’s age, highest school grades of caregiver, highest school grade of household head, household size, financial support, socio-economic status, and home stimulation score. | | | | | | |
| IDELA = International Development and Early Learning Assessment; CBCL = Child Behavior Checklist; CI = Confidence interval | | | | | | |
