# Supplementary material for: Assessing the association between air pollution and child development in São Paulo, Brazil
Source: PLoS One. 2022 May 13;17(5):e0268192. doi: 10.1371/journal.pone.0268192 (PMC9106172; doi:10.1371/journal.pone.0268192)
Supplement: Attachment — Submitted filename: Response to Reviewers.docx [file pone.0268192.s006.docx]

Dear Dr. Rodrigues Da Silva, dear Dr. Baltar,

We appreciate the opportunity to revise our manuscript and would also like to thank you for giving us more time to make these changes. Below you will find our detailed responses to each arisen point.

1. We adapted the style of the manuscript following PLOS ONE requirements.
2. With the manuscript, we are also submitting the minimal data set as Supporting Information file (S4 Dataset).
3. We added the following key information to the Methods section: *The data collection for the SP-ROC cohort was approved by the Hospital das Clínicas at the University of São Paulo ethics committee (CAPPESQ HC_FMUSP) under protocol Nº 01604312.1.0000.0065. Written informed consent from the caregivers was obtained before data collection. The work presented in this paper was formally reviewed and approved by the Ethikkommission Nordwest- und Zentralschweiz (EKNZ) under protocol N° AO_2020-00024.*
4. We decided to remove the figure 2, which was not very informative.
5. We added the captions fort the Supporting Information at the end of the manuscript and we adapted the in-text citations accordingly.

Dear reviewers,

Many thanks for your careful review and helpful comments. We have done our best to address all of them as described in further detail below.

Reviewer #1: This work is a useful addition to the literature. However, there are some instances where the author needs to make some improvements. Below are my comments:

1) The authors should highlight the methodology's appropriateness. Why linear regression model was used? I have doubts that this is the most appropriate statistical approach. Moreover, in Methods section, it’s important to describe how the model and metrics were applied, including their equations.

Thank you for this comment and apologies for the lack of clarity in the original manuscript. The linear model was used because we are working with a normalized outcome variable, i.e. z-score with mean 0 and SD 1. In the text we added following sentence to clarify the appropriateness of the method: “*We standardised both outcomes to mean zero and standard deviation one.” Line 146-147.* We also added the following equations to the text, which describe the exact model used in the regressions.

$$Y_{i}= \beta_{0} + \beta_{1}{NO}_{2} + \varepsilon_{i}$$

$$Y_{i}= \beta_{0} + \beta_{1}{NO}_{2} + \beta_{2}\mathrm{gender}+ \beta_{3}age\_months+ \beta_{4}\mathrm{skincolor}+ \beta_{5}\mathrm{birthweight}+ \beta_{6}gest\_length+ \beta_{7}\mathrm{delivery}+ \beta_{8}age\_mother+ \beta_{9}mother\_skincolor+ \beta_{10}mother\_depression+ \beta_{11}caregiver\_age+ \beta_{12}marital\_status+ \beta_{13}caregiver\_relation+ \beta_{14}caregiver\_grade+ \beta_{15}headhousehold\_grade+ \beta_{16}household\_size+ \beta_{17}financial\_support + \beta_{18}\mathrm{SES}+ \beta_{19}\mathrm{MICS}+ \varepsilon_{i}$$

$2) The influence of meteorological conditions on the air pollutant concentrations needs to$be considered at the time of the study.

Many thanks for this comment. We took differences in meteorological conditions in our air pollution modelling into account by performing the NO_2_ monitoring campaigns in 2 different seasons; winter (August 2019) reflecting hot, humid and rainy conditions and summer (Feb 2019) reflecting the dry conditions. At the same time we measured NO_2_ continuously (weekly NO_2_ measurements) at a regional background site inside the study area over a full year. Using this continuous record, we were able to adjust the summer and winter NO_2_ measurements from the 2 campaigns to an adjusted annual average NO_2_ concentration. The details of the monitoring campaign and the subsequent calculation of the adjusted annual average NO_2_ concentrations are described in Luminati et al. (2021). In the revised text we integrated this comment on lines *112-115*:

*“The first measurements were done in summer (February), reflecting the hot humid and rainy conditions, the second in winter (August) reflecting the colder and dry conditions. Temporal adjustments were made using weekly data collected at a reference monitoring site all year around to calculate adjusted annual mean NO2 concentrations.”*

3) Discussion: comparisons with previous studies are absent.

We carefully edited the discussion.

Reviewer #2: The present study addressed the association between nitrogen dioxide exposure and children’s cognitive and behavioral development in São Paulo City. It is a relevant theme and the importance of the study is clearly stated in the manuscript. However, a substantial modification is required and some issues must be addressed:

Many thanks for these friendly comments. We did our best to address all remaining issues as outlined below.

I suggest to include more information regarding the NO_2_ when the authors introduced the air pollutants, considering it is the pollutant included in the study. Why did the authors choose to monitor the NO_2_ concentration and why the concentration of other pollutants was not included?

We focused on NO_2_ as this is a good proxy for traffic related air pollution and it is relatively easy and cheap to monitor. We have added this information to the text on lines 107-109 where we write:

*“With the goal to investigate the effect of air pollution on child development, we focused on NO2 as our primary pollutant. NO2 primarily results from traffic related burning of fossil fuel and can be measured relatively easily and cheaply using passive gas samplers (80).“*

I suggest a reorganization of the introduction section. Some paragraphs could be merged to make the text more concise.

We did some changes in the introduction in order to make the text more concise.

The characterization of the study area could be more specific, including the main pollution sources nearby and a figure to illustrate.

*We added some more information in the text addressing this comment as suggested. On lines 84-87 of the revised manuscript, we write:*

*“The study area is characterized by a dense network of streets of all sizes and includes a wide variety of different neighbourhoods, ranging from the university campus, residential neighbourhoods and green areas to favelas and industrial areas (29).”*

Why did the authors choose a 2 one-week monitoring period in the summer and winter, This information could be better explained in the methods section.

*Thank you. We integrated more information in the revised text on lines 112-115, where we write: ”The first measurements were done in summer (February), reflecting the hot humid and rainy conditions, the second in winter (August) reflecting the colder and dry conditions.”*

To continue the revision process of this article, the authors should state clearly the contribution to the results to the literature, make a more robust discussion about their data, and demonstrate how the strengths of the study minimize the limitations observed. The author needs to rearrange the conclusions, once the information in the conclusion is still not very informative and do not reflect the content and results of the research that has been done.

*We carefully edited the discussion and conclusion.*
