# Supplementary material for: Assessing the association between air pollution and child development in São Paulo, Brazil
Source: PLoS One. 2022 May 13;17(5):e0268192. doi: 10.1371/journal.pone.0268192 (PMC9106172; doi:10.1371/journal.pone.0268192)
Supplement: Attachment — Submitted filename: Response to Reviewers 16 March 2022.docx [file pone.0268192.s007.docx]

**Reviewer #2 Comments:**

***“The authors made the modifications in the introduction, however, the discussion is still insufficient to make the result more robust and it is not well explored. The authors had an interest and complete databases, however, they could not discuss the absence of result and attributed only to limitations, as the lack of data and sample.”***

(Reply): Thanks for the kind review. We are happy to hear that the introduction is good shape now. We have done our best to further improve the Discussion. We agree that we have a rather nice and comprehensive database, but that unfortunately does not allow us to directly identify the reason why we do not see the expected outcome gradient. There are many potential reasons why this could be the case, and we can only speculate about them. We pasted the revised Discussion below and would be grateful for any further suggestions.

“Despite these high levels of exposure, we found no association between exposure to NO_2_, and children’s cognitive development or children’s behavioural problems in our sample.

In the literature, evidence on the association between NO_2_ and child development remains uncertain. Freire et al. found a negative association between NO_2_ exposure higher than 24 μg/m^3^ and general cognition compared with NO_2_ exposures lower than 15.4 μg/m^3^ (37), although these differences were not statistically significant. Forns et al. looked at air pollution in different schools in Barcelona and found a significant negative association with all measured pollutants and working memory, and largest associations for NO_2_ (13). Comparing children at high and low NO_2_ exposure Sunyer et al. found negative associations between NO_2_ and working memory (14). In general, most of the literature seems to find a negative relationship between NO_2_ exposure and children’s cognition, although the overall evidence was classified as insufficient in literature review from 2015 (38). The same literature review also reported insufficient evidence for the association between NO_2_ and child behaviour (38). The majority of studies published so far have focused on exposure levels at school (13, 14, 39).

This project was initiated with a strong prior of finding negative associations between NO_2_ exposure and children’s development and behaviour. We performed NO2 measurements directly in the study area, within the personal premises of 80 SP-ROC study participants. These measurements enabled us to build a robust LUR model to predict NO2 concentrations. Another strength of our study is the SP-ROC-Cohort dataset, which contains detailed information on families and home environments, enabling us control for a large range of potential confounders. The data set also contains both direct assessments of children’s cognitive skills and detailed reports on behavioural issues, allowing us to look at both dimensions of child development.

Despite this setup, we found no evidence of negative associations between NO_2_ and child’s development and behaviour. There are some limitations that may have contributed to the lack of association. First, we estimated the NO_2_ concentration at the residential address of the children, which describes their exposure only during a part of the day. Most of the children in the study went to kindergarten or school, and we did not collected data on NO_2_ concentration in these locations. Second, as with all NO_2_ exposure models, a certain extent of misclassification of exposure levels cannot be avoided. Given that we measured exposure levels twice across 80 different measurement locations, the scope for such measurement error seems however small. From a purely statistical perspective, we were of course also limited by the very high average exposure levels with relatively limited variability. The sample contained only 2 children with NO_2_ exposure levels below 35 μg/m^3^, and only a few with NO_2_ exposure levels above 50 μg/m^3^. Even if NO_2_ exposure is harmful, it is possible that differences in exposure levels in our sample were too small to result in statistically detectable differences in child outcomes. It is of course also possible that the true causal effect of NO_2_ exposure is smaller than the current literature suggests, or that negative effects only occur in settings where other protective factors (that may have been in place here) are not present.

Further research will be needed to better understand the diverging results in the current literature.”
